# DNA virome of ticks in the Northeast and Hubei provinces of China reveals diverse single-stranded circular DNA viruses

**DOI:** 10.1186/s13071-023-05684-6

**Published:** 2023-02-09

**Authors:** Yuhang Liu, Lei Guo, Guoshuai Wang, Fei Gao, Zhongzhong Tu, Deming Xu, Lanshun Sun, Le Yi, Guoqiang Zhu, Changchun Tu, Biao He

**Affiliations:** 1 Jiangsu Co-innovation Center for Prevention and Control of Important Animal Infectious Diseases and Zoonosis, Yangzhou University, Yangzhou, Jiangsu China; 2 Changchun Veterinary Research Institute, Chinese Academy of Agricultural Sciences, Changchun, Jilin China; 3 Division of Wildlife and Plant Conservation, State Forestry and Grassland Administration, Changchun, Jilin China; 4 Section of Wildlife Conservation, Greater Xing’an Mountains Forestry Group Corporation, Jiagedaqi, Heilongjiang China; 5 Forestry Bureau of Linjiang City, Linjiang, Jilin China; 6 Provincial Wildlife Disease Monitoring Station of Shuanghe, Xunke, Heilongjiang China

**Keywords:** Ticks, DNA virome, CRESS DNA viruses, Genetic diversity

## Abstract

**Background:**

Ticks are medically important vectors capable of transmitting a variety of pathogens to and between host species. Although the spectrum of tick-borne RNA viruses has been frequently investigated, the diversity of tick-borne DNA viruses remains largely unknown.

**Methods:**

A total of 1571 ticks were collected from forests and infested animals, and the diversity of the viruses they harbored was profiled using a DNA-specific virome method. The viromic data were phylogenetically analyzed and validated by PCR assays.

**Results:**

Although diverse and abundant prokaryotic viruses were identified in the collected ticks, only eukaryotic DNA viruses with single-stranded circular genomes covering the anelloviruses and circular replication-associated (Rep) protein-encoding single-stranded (CRESS) DNA viruses were recovered from ticks. Anelloviruses were detected only in two tick pools, but CRESS DNA viruses were prevalent across these ticks except in one pool of *Dermacentor* spp. ticks. Phylogenetic analyses revealed that these tick-borne CRESS DNA viruses were related to viruses recovered from animal feces, tissues and even environmental samples, suggesting that their presence may be largely explained by environmental factors rather than by tick species and host blood meals.

**Conclusions:**

Based on the results, tick-borne eukaryotic DNA viruses appear to be much less common than eukaryotic RNA viruses. Investigations involving a wider collection area and more diverse tick species are required to further support this speculation.

**Graphical Abstract:**

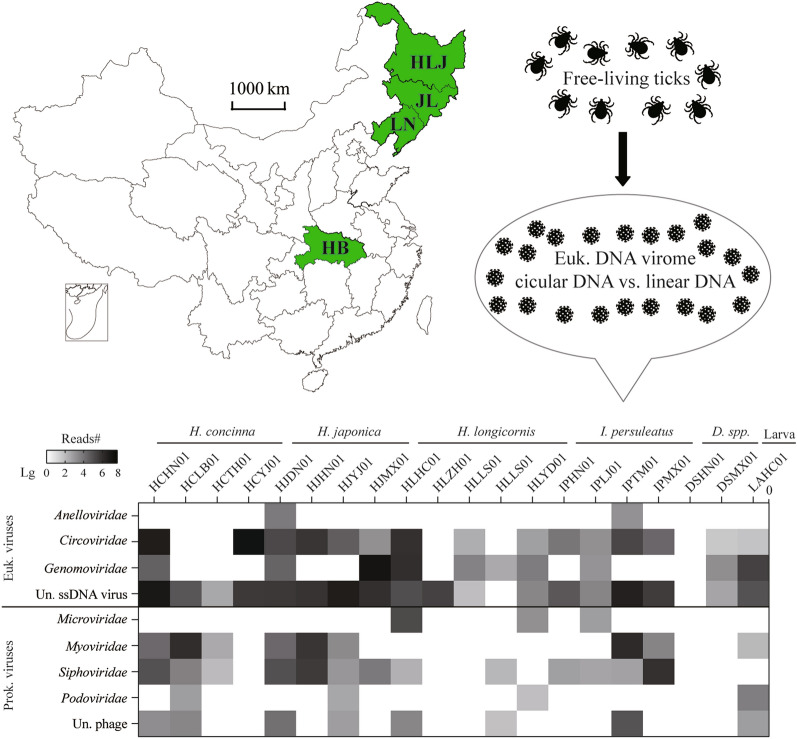

## Background

Ticks are among the most important arthropod vectors capable of transmitting a wide range of microorganisms in humans, wildlife and domestic animals, including viruses, bacteria, protozoa, fungi and nematodes [1]. Due to their obligate propensity for feeding on the blood of different host species, ticks maintain the complex circulation of tick-borne pathogens in vertebrate hosts, seriously hindering the control and prevention of tick-borne diseases (TBDs) [2]. The changes in climate that are currently ongoing have significantly impacted the life-cycle of ticks, resulting in lower mortality during overwintering and a longer active period [3]. In addition, because the interfaces between tick habitats and human settlements are frequently disturbed by human activity [4], TBDs, especially those caused by a viral agent, are increasing; for example a number of new tick-borne viruses (TBVs) have been identified as causative agents of human febrile illnesses in northeast (NE) China over the past few years [5–8]. Therefore, continuous investigation and surveillance of genetically diverse TBVs is of significant importance for the prevention and control of emerging TBDs.

TBVs were first identified more than a century ago, but most studies have been confined to RNA viruses that are classified into two orders, eight families and at least 11 genera. It is known that lethal TBDs are predominantly caused by TBVs that are RNA viruses (RNA TBVs). For example, tick-borne encephalitis (TBE) and Omsk hemorrhagic fever are caused by flaviviruses [9, 10], while Crimean-Congo hemorrhagic fever virus (CCHFV) and severe fever with thrombocytopenia syndrome virus (SFTSV) are responsible for the deadly hemorrhagic fever diseases [11, 12]. As a result, RNA TBVs have received more attention, with tick viromic profiling focusing primarily on RNA viruses [13, 14]. Conversely, TBVs that are DNA viruses (DNA TBVs) have been relatively neglected, even though two well-known DNA viruses, namely African swine fever virus (ASFV) and lumpy skin disease virus (LSDV), are associated with ticks and cause serious diseases with significant economic consequences in the livestock industry. The former is transmitted by soft ticks from the genus *Ornithodoros* [15], while the latter belongs to the genus *Capripoxvirus* of the family *Poxviridae* [16] and is transmitted by *Rhipicephalus appendiculatus* and *Amblyomma hebraeum* ticks [17]. In addition, a few studies have identified several distinct small DNA viruses, such as circoviruses from *Haemaphysalis longicornis* and *Haemaphysalis crenulatus* ticks [18], genomoviruses and anelloviruses from *Dermacentor variabilis* and *Ixodes scapularis* ticks [19] and parvoviruses from *Ixodes ricinus* and *Rhipicephalus sanguineus* ticks [20, 21]. Also, murine gammaherpesvirus 68, a natural pathogen of murid rodents, was also identified in *Dermacentor reticulatus* and *I. ricinus* ticks, which has expanded the spectrum of DNA TBVs to the family *Herpesviridae* [22, 23].

The sporadic studies carried out on DNA TBVs are insufficient to capture a complete view of the diversity of DNA TBVs. To further understand the DNA viral spectrum harbored by ticks, we used a DNA-specific virome method to investigate the DNA virome harbored by free-living and engorged ticks collected from the NE province and Hubei province in China, respectively. The method used here employs the multiple displacement amplification (MDA) technique to magnify viral DNA and can capture DNA viruses of all genome types [24–26]. The two regions of this study are known to be natural habitats of abundant ticks and hotspots of many TBDs, such as TBE and SFTS [27–29]. The results revealed diverse tick-borne small circular DNA viruses, providing insight into the diversity of DNA TBVs.

## Methods

### Tick sampling and species identification

**Fig. 1:**
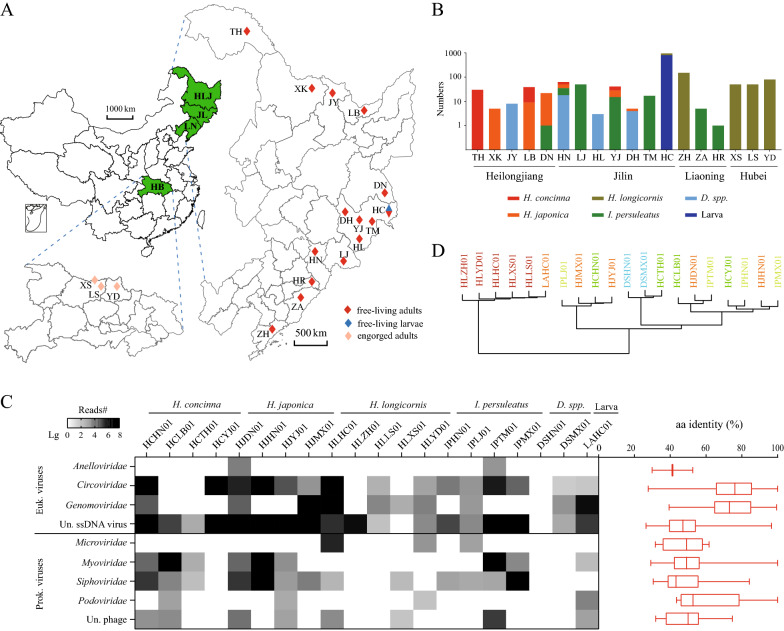
Sample information and virome overview. **a** Geographical distribution of tick sampling sites. HLJ, Heilongjiang province; JL, Jilin province; LN, Liaoning province; HB, Hubei province; TH, Tahe; XK, Xunke; JY, Jiayin; LB, Luobei; DN, Dongning; HN, Huinan; LJ, Linjiang; HL, Helong; YJ, Yanji; DH, Dunhua; TM, Tumen; HC, Hunchun; ZH, Zhuanghe; ZA, Zhenan; HR, Huanren; XS, Xinshi; LS, Liusheng; YD, Yindian. **b** Numbers of ticks sampled at each site. **c** Overview of tick DNA virome. Left panel: Heat map of viral reads from 20 libraries. Right panel: amino acid identity range of these virus-like contigs with reference sequences in Genbank. Each box plot illustrates the estimated median (center line), and upper and lower quartiles (box limits) of the similarity. The identifier of the library was composed of four letters and a two-digit number, whereby the first two letters are abbreviations for tick species, the next two letters are indicative of the sampling sites and the number refers to the pool’s number. Euk, Eukaryotic; Prok, prokaryotic; Un, unclassified. **d** K-mer distance-based clustering of these pools. Pools with the same tick species are identified using the same color

The most favorable climatic conditions for tick proliferation are during the spring and summer. Consequently, we collected ticks by dragging and flagging vegetation in forests adjacent to human settlements in Heilongjiang, Jilin and Liaoning provinces between May and June 2021. We also directly plucked ticks from infested goats in Hubei Province in May 2016 (Fig. 1a, b). Ticks were placed immediately upon collection in labeled vials and transported on dry ice to our laboratory. Adult ticks were initially identified to the species level using tick taxonomic keys by a trained expert under a stereo microscope (Nikon model H550S; Nikon Corp., Tokyo, Japan) based various morphological characters, such as the scutum, the basis capitula and palp, the anal groove and the shape and size of the spurs on the coxae [30–33]. Species identification was further confirmed by sequencing the mitochondrial (mt) cytochrome* c* oxidase subunit I gene (*COI*) [34], which was also used to identify the species of larval ticks.

### Sample pretreatment and high-throughput sequencing

All ticks were grouped into pools according to sampling locations and species, with each pool containing 13–150 adult ticks or 800 larvae (Fig. 1c). Each pool’s name provides information on tick species (the first 2 letters), location (the second 2 letters) and pool number (the last 2 numbers). Ticks in each pool were first washed 3 times with sterile phosphate-buffered saline solution (PBS; pH = 7.4) and then homogenized using a ground-glass tissue grinder in 1 ml of sterile magnesium salt buffer (50 mM Tris, 10 mM MgSO_4_, 0.1 M NaCl, pH 7.5). Homogenates were centrifuged at 8000 *g* for 10 min at 4 °C to remove debris. The supernatants were filtered through a Millex filter (pore size: 0.45 μm) (MilliporeSigma, Burlington, MA, USA) and digested with 1 μl DNase I (TaKaRa, Dalian, China) and 1 μl RNase A (TaKaRa) at 37 °C for 60 min to eliminate host genomic DNA and other free nucleic acids. The total DNA was then extracted immediately using a DNeasy Blood & Tissue Kit (Qiagen, Hilden, Germany) according to the manufacturer’s protocol. The DNA-specific MDA method was employed to amplify the DNA using an Illustra GenomiPhi V2 DNA Amplification Kit (GE Healthcare, Chicago, IL, USA). A 1-μg sample of products was subjected to DNA library preparation using a NEB Next® Ultra™ DNA Library Prep Kit for Illumina (NEB, Ipswich, MA, USA), followed by paired-end (150-bp) sequencing on a NovaSeq sequencer (Illumina, Inc., San Diego, CA). At least 6 Gb data of each library were generated. Cross and foreign contamination are common in metagenomic sequencing [35]; six pools of bat rectums and double-distilled water were also processed synchronously to determine potential contamination events because ticks and bats belong to different classes and are thought to harbor distinct viromes [35].

### Annotation and comparison of virome

The fastp v0.19.7 tool was used for the initial quality control of the raw data. Host sequences were removed from clean data by mapping against the whole genomic assemblies of *H. longicorni* (accession: GCA_013339765.2), *Ixodes persulcatus* (accession: GCA_013358835.1) and *Dermacentor silvarum* (accession: GCA_013339745.1) using the Bowtie2 v2.4.1 tool. Bacterial, archaeal and fungal reads in the remaining data were further subtracted using a fast taxonomic classification with Kraken2 (v2.0.9-beta) and a custom RefSeq-based database. The unclassified reads were de novo assembled separately using the metaSPAdes v3.14.9 metagenome assembler, and circular genomes were recovered using Recycler. Prior to annotation, tick viromic contigs were compared to those of bats using BLASTn. If an alignment was achieved with length > 500 bp and identity > 99%, the tick contig was considered to be a contaminant. This comparison procedure detected hundreds of tick contigs closely related to bat contigs. These sequences were finally validated to be eukaryotic sequences or genomic fragments of DNA viruses with low confidence after querying Genbank and, therefore, were deleted from the assembly. However, because the double-distilled water did not result in any nucleic acid products, no further action was taken. The remaining contigs were retained for BLASTn (v2.10.0) and diamond BLASTx (v0.9.35) searches against our refined eukaryotic viral reference database (EVRD)—nucleotides/amino acids (nt/aa) [35]. To investigate prokaryotic viruses, we also searched these contigs against the bacteriophage aa sequences of UniProt database. The read abundance was counted by mapping unclassified reads back to virus-like contigs using bowtie2 and SAMtools v1.10. To examine the viromic difference between libraries, we compared the unclassified data sets using MASH v2.2. A sketch file of each data set was created with sketch size of 10,000 and K-mer size of 21. These sketches were mutually compared to generate the MASH distances, based on which Ward’s minimum variance clustering was performed in the R environment v4.1.3 ® Foundation for Statistical Computing, Vienna, Austria).

### PCR validation

The completeness of circular genome representatives was validated by PCR using partially overlapped back-to-back primer pairs. The DNA of the remaining supernatants of a particular pool was extracted as described above, from which the circular genome was recovered by PCR with Phanta® Max Super-Fidelity DNA Polymerase (Vazyme, Wuhan, China) and primer pairs with the following thermocycler program: pre-denaturation at 98 °C for 1 min; 35 cycles of denaturation at 98 °C for 5 s, annealing at 59 °C for 10 s and extension at 72 °C for 10 s; and a final extension at 72 °C for 5 min. The products were examined by electrophoresis in a 1% agarose gel with the expected products then directly sequenced on an ABI 3730xl DNA analyzer (Applied Biosystems, Thermo Fisher Scientific, Waltham, MA, USA). The sequence was further aligned against the initial circular genomes using MegAlign to check its accuracy.

### Phylogenetic analyses

The putative open-reading frames (ORFs) of circular virus genomes were identified using NCBI ORF Finder (http://www.ncbi.nlm.nih.gov/gorf/gorf.html). References of the replication-associated protein genes (*Rep*) were withdrawn from Genbank after BLASTx search and aligned together with the *Rep* aa sequences identified here using the MAFFT L-INS-I algorithm. The alignments were used to infer maximum likelihood phylogenetic trees using IQ-TREE with the LG + G substitution model selected using ModelFinder. The resulting phylogenetic trees were mid-point rooted, and branches with < 70% bootstrap support were collapsed using the TreeGraph 2 graphical editor for phylogenetic trees.

## Results

### Overview of tick-borne DNA virome

A total of 1571 ticks were collected from 18 locations in Heilongjiang, Jilin, Liaoning and Hubei provinces (Fig. 1a, b). Ticks from the first three provinces, all located in NE China, were all free-living and comprised adults (*n* = 591) and larvae (*n* = 800), while the ticks collected from Hubei Province were adults plucked from infested goats (*n* = 180) (Fig. 1a, b). These ticks were identified as *Haemaphysalis concinna* (*n* = 86), *Haemaphysalis japonica* (*n* = 864, including 800 larvae), *H. longicornis* (*n* = 480), *I. persulcatus* (*n* = 108) and *Dermacentor* spp. (*n* = 33) (Fig. 1b). They were grouped into 20 pools based on species and sampling location and subjected to DNA virome profiling, which revealed 875 virus-like contigs with 20 complete circular genomes. All 875 contigs were annotated to small circular DNA viruses, i.e. *Anelloviridae*, *Circoviridae*, *Genomoviridae* and unclassified viruses with single-stranded circular DNA (sscDNA) (Fig. 1c). The annotation using UniProt also uncovered diverse bacteriophages of different genomic types, including those with double-stranded linear and single-stranded circular genomes (Fig. 1c). These showed a wide range of aa identities to the reference sequences, with the majority distantly related to currently known viruses (Fig. 1c). Mapping of the reads back to contigs showed that virus-like reads composed 0 (DSHN01) to 54.5% (HCYJ01) of their total reads (Fig. 1c). *Circoviridae* and unclassified sscDNA viruses were widely distributed in these libraries with the highest abundances, while anelloviruses were only detected in two libraries with 0.9% and 0.1% relative abundances in HJDN01 and IPTM01, respectively (Fig. 1c). Interestingly, the larva pool LAHC01 also contained 1.6% reads related to *Circoviridae*, *Genomoviridae* and unclassified ssDNA viruses (Fig. 1C). Multiple tick species were involved in this study, providing an ideal model to inspect the DNA viromic composition feature of tick species. We abstracted the unassigned data into K-mer sketches using MASH and clustered these libraries based on the K-mer distances. Although one cluster was primarily composed of pools of *H. longicornis* ticks regardless of their sampling site or diet (Fig. 1d), the clustering of the remaining tick pools was not significantly correlated with tick species (Fig. 1d), implying that host species have little influence on the sequence composition feature of the tick DNA virome.

### Characterization of circular Rep protein-encoding DNA molecules

**Fig. 2:**
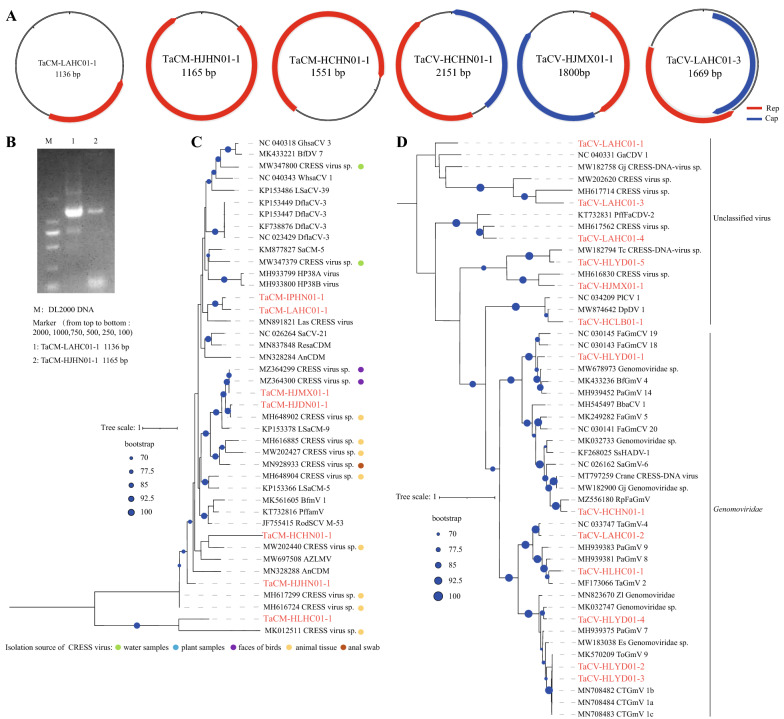
Genomic characterization of TaCMs and TaCVs. **a** Genomic structures of three TaCM and three TaCV representatives. **b** Back-to-back PCR validations of TaCMs LAHC01-1 and HJHN01-1. **c** Phylogenetic analysis of 7 TaCMs (highlighted in red) with their genetic neighbors retrieved from Genbank based on Rep proteins. **d** Mid-point rooted maximum likelihood phylogenetic tree of 13 TaCV Rep proteins (highlighted in red). Rep, Replication-associated protein; TaCVs, tick-associated circular DNA viruses; TACMs, tick-associated circular DNA molecules

Among the obtained 20 circular genomes, seven were 1105–1551 nt in length and only encoded a Rep protein (Fig. 2a). PCR amplification using partially overlapped back-to-back primer pairs and sequencing confirmed their circular structures (Fig. 2b). We termed these genomes tick-associated circular DNA molecules (TaCMs). The putative Rep proteins of these TaCMs were 101–309 aa long and showed a wide range of similarities to known sequences. Phylogenetic analyses showed that these Rep sequences were clustered into five clades within two major branches. Although circular DNA molecules in Genbank were among the genetic neighbors of some TaCMs, the closest relatives were all circular Rep-encoding single-stranded (CRESS) DNA viruses from a variety of sources, such as plants, bird feces and animal tissues (Fig. 2c). Most TaCM *Rep* were very divergent from their references, with 29.0–78.5% aa identities, whereas TaCM HJMX01-1 was highly similar to CRESS DNA viruses recovered from fecal metagenomes of Australian birds with up to 100% aa identities.

### Genetic diversity of circular DNA viruses

The remaining 13 circular genomes were 1669–2535 nt long and had the typical structure of circovirus, i.e. encoding Rep and catabolite activator proteins (Cap) (Fig. 2a). The two genes in most genomes were bi-directionally oriented, either detaching from each other or overlapping at their C-terminals (Fig. 2a). However, there were two genomes with uni-directionally oriented *Rep* and *Cap* (Fig. 2a), which were further verified by PCR amplification. These tick-associated circular DNA viruses (TaCVs) were distantly related to each other and showed low identities to known viruses (Fig. 2d). Phylogenetic analyses based on Rep proteins showed that these TaCVs were classified into the family *Genomoviridae* and unclassified viruses (Fig. 2d). Within the clade of *Genomoviridae*, the seven TaCVs genetically neighbored to genomoviruses (≥ 66.4% aa identities) originating from a wide range of samples, such as red panda feces (RpfaGmV), air-borne particulate matters (BfGmV 4 and PaGmV 14) and even citrus (CTGmV) (Fig. 2D). The highest similarity was observed for TaCV HLYD01-2/3, approximately 97% aa identical to CTGmVs. It should be noted that TaCV HLHC01-1 was 85.4% identical to TaGmV 2, a genomovirus recovered from an American *Dermacentor variabilis* tick. These TaCVs within the clades of unclassified viruses showed highly genetic diversities with each other and formed polyphyletic clades with CRESS DNA viruses from different hosts with 35.5–77.5% aa identities.

## Discussion

Tick-borne diseases pose a significant threat to public health and are mostly caused by infection with RNA viruses. Hence, RNA TBVs are often prioritized in investigations using either pathogen-specific or viromic methods, with the result that current knowledge of tick-borne RNA viruses is adequate. By contrast, studies on the diversity of DNA TBVs have been largely neglected. The tick-borne DNA virome in the present study revealed genetically diverse sscDNA viruses. The method used here is known to over-amplify small circular DNA genomes [25], but it also has an excellent capability to capture linear DNA genomes [26]. The presence of linear DNA bacteriophages highly implied that these ticks harbored rare linear eukaryotic DNA viruses. Viruses detected in ticks can be divided into tick-borne viruses that replicate in tick organs and viruses that are simply mechanically carried by ticks via feeding. The ticks collected from NE China were unfed and collected directly from the natural mixed forests. The detection of these viruses also in the tick larvae, suggests that the virome profiled here should be explained by tick-associated or environmental factors rather than host blood meals. The genetic diversity of a virus can be generally be partially shaped by its host species. For example, SFTSV is mainly maintained by *H. longicornis* [36], while CCHFV is associated with *Hyalomma* spp. [37]. However, the sscDNA viruses detected in the present study did not show any specificity to tick species, with some even having a close genetic relationship with viruses recovered from plants and animal feces. Hence, it is reasonable to infer that these tick-borne CRESS DNA viruses are associated primarily with environmental factors, rather than with viruses that actively replicate in ticks.

CRESS DNA viruses are among the most intriguing of viruses due to their ubiquity and diverse host range [38]. Their homologous Rep proteins facilitate the rolling-circle replication of genomes [39]. The number of known CRESS DNA viruses has greatly increased in recent years, largely due to the wide application of viral metagenomics. The latest International Committee on Taxonomy of Viruses (ICTV) taxonomy release groups CRESS DNA viruses into 11 families within the order *Cressdnavircota*, including the animal-associated *Circoviridae*, the plant-associated *Geminiviridae*, *Metaxyviridae* and *Nanoviridae*, the fungal-associated *Genomoviridae*, the diatom-associated *Bacilladnaviridae*, the protist-associated *Naryaviridae*, *Nenyavirdae* and *Vilyaviridae*, as well as two families with unknown host range: *Smacoviridae* and *Redondoviridae* [40, 41]. Interestingly, the *Rep* of CRESS DNA viruses is also associated with the sole protein encoded by a large number of circular, single-stranded DNA molecules. These molecules are 1.0–1.4 kb in length and have been assigned to the family *Alphasatellitedae* [42]*.* Of note is that all TaCMs identified in the present study were encompassed by CRESS DNA viruses, implying that TaCMs might originate from either defective genomes or small genome components of CRESS DNA viruses with multipartite genomes [43]. Most sequences recovered here were highly divergent from known references, with some capable of being classified as new species. These results suggest that tick-associated CRESS DNA viruses are much more diverse and abundant than expected.

## Conclusion

This study provides important data to understand the spectrum of DNA viruses harbored by ticks in the NE provinces and Hubei Province of China. Although the ticks analyzed here were collected from a wide range of locations, covering five species with more than 1000 individuals, we did not find any linear DNA eukaryotic viruses, highly implying that the latter are only rarely harbored by ticks in these regions. However, considering that some DNA TBVs, such as ASFV and LSDV, pose severe threats to animal health, their surveillance should not be neglected but rather emphasized, similar to surveillance for RNA TBVs.

## Data Availability

All sequence reads generated in this study are available in the NCBI SRA database under BioProject accession PRJNA843368. The assembled complete genomes have been deposited in GenBank under accession numbers ON668986 to ON669005.

## Abbreviations

aa: Amino acid
CRESS: Circular Rep-encoding single-stranded
EVRD: Eukaryotic Viral Reference Database
ICTV: International Committee on Taxonomy of Viruses
ORF: Open reading frame
sscDNA: Single-stranded circular DNA
Rep: Replication-associated protein
TaCMs: Tick-associated circular DNA molecules
TaCVs: Tick-associated circular DNA viruses
TBD: Tick-borne disease
TBV: Tick-borne virus
